# Supplementary material for: Advanced Silicon Modeling of Native Mitral Valve Physiology: A New Standard for Device and Procedure Testing
Source: Bioengineering (Basel). 2025 Apr 7;12(4):397. doi: 10.3390/bioengineering12040397 (PMC12024820; doi:10.3390/bioengineering12040397)
Supplement: Supplementary file 1 [file bioengineering-12-00397-s001.zip › bioengineering-3480026-supplementary.pdf]

SUPPLEMENTARY DATA

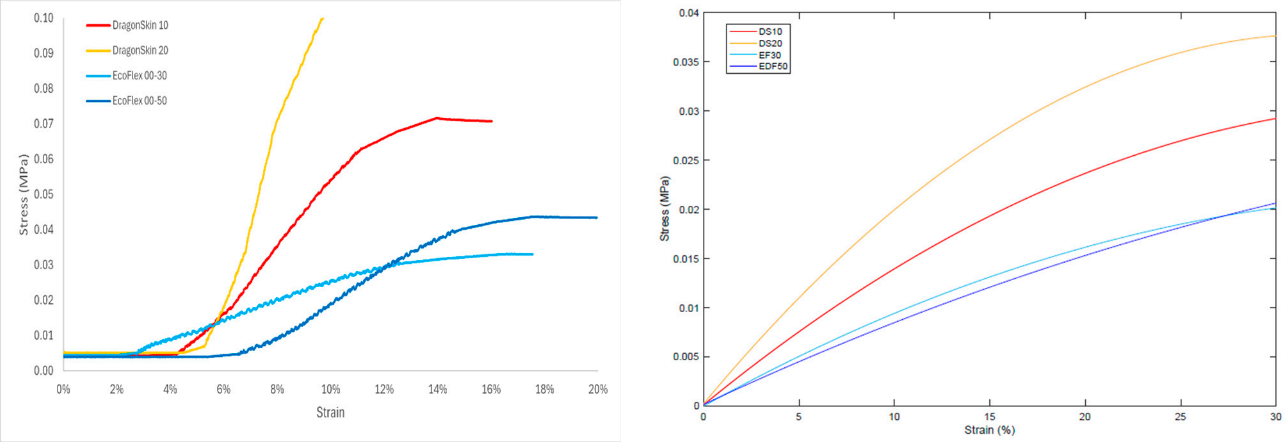

Figure S1. Stress-Strain curves obtained from biaxial testing (left pannel) and uniaxial testing (right pannel) of tested silicons rubbers.

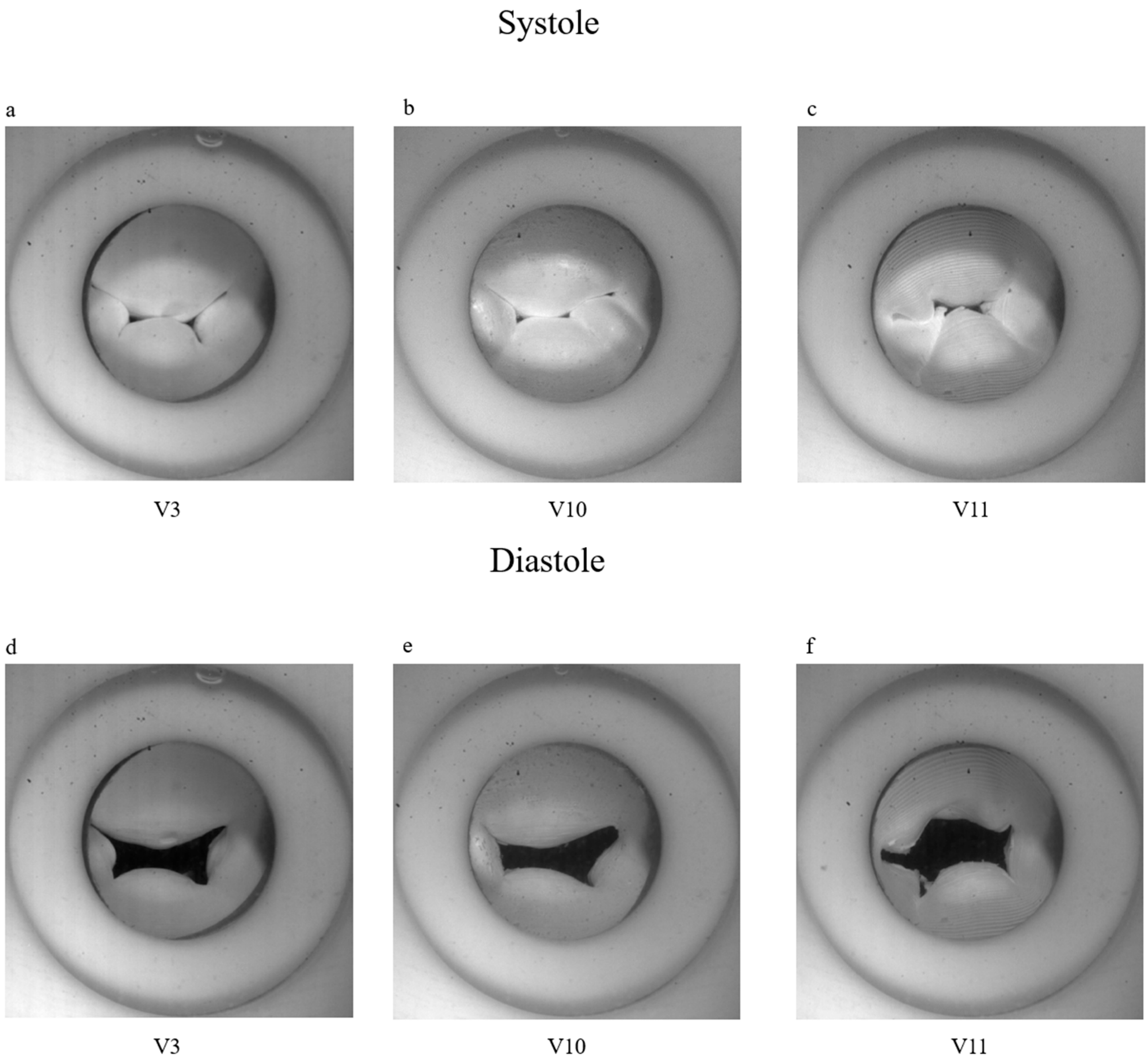

Figure S2. Stenotic-like behavior induced by V3 (a,d), V10 (b,e) and V11 (c,f).

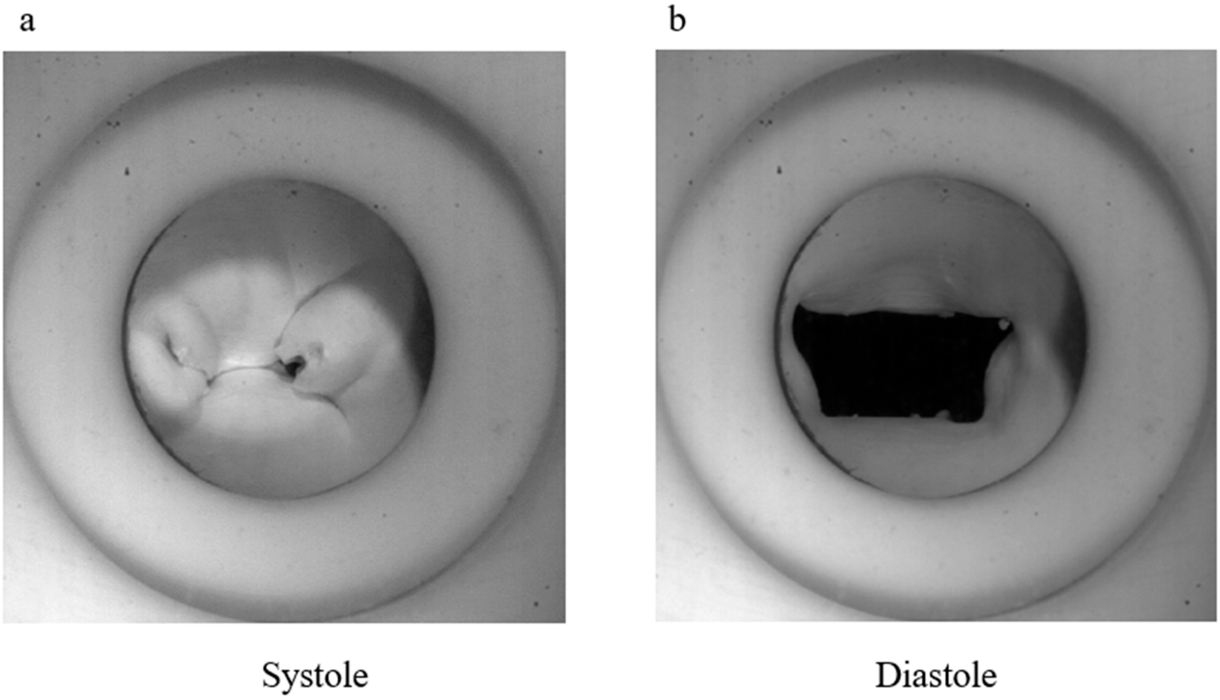

Figure S3. Fibroelastic-like deficiency behavior induced by V1.
